# NABat: A top-down, bottom-up solution to collaborative continental-scale monitoring

**DOI:** 10.1007/s13280-020-01411-y

**Published:** 2021-01-17

**Authors:** Brian E. Reichert, Mylea Bayless, Tina L. Cheng, Jeremy T. H. Coleman, Charles M. Francis, Winifred F. Frick, Benjamin S. Gotthold, Kathryn M. Irvine, Cori Lausen, Han Li, Susan C. Loeb, Jonathan D. Reichard, Thomas J. Rodhouse, Jordi L. Segers, Jeremy L. Siemers, Wayne E. Thogmartin, Theodore J. Weller

**Affiliations:** 1 U.S. Geological Survey Fort Collins Science Center, Fort Collins, CO USA; 2 Bat Conservation International, Austin, TX USA; 3 U.S. Fish and Wildlife Service, Hadley, MA USA; 4 Canadian Wildlife Service, Environment and Climate Change Canada, National Wildlife Research Centre, Ottawa, ON Canada; 5 Department of Ecology and Evolutionary Biology, University of California, Santa Cruz, CA USA; 6 U.S. Geological Survey Northern Rocky Mountain Science Center, Bozeman, MT USA; 7 Wildlife Conservation Society Canada, Kaslo, BC Canada; 8 Department of Biology, University of North Carolina Greensboro, Greensboro, NC USA; 9 USDA Forest Service, Southern Research Station, Clemson, SC USA; 10 National Park Service Upper Columbia Basin Network, Bend, OR USA; 11 Canadian Wildlife Health Cooperative, Charlottetown, PEI Canada; 12 Colorado State University, Fort Collins, CO USA; 13 U.S. Geological Survey Upper Midwest Environmental Sciences Center, Lacrosse, WI USA; 14 USDA Forest Service, Pacific Southwest Research Station, Arcata, CA USA

**Keywords:** Bats, Collaborative monitoring, Master sample, NABat, Sample design, White-nose syndrome

## Abstract

Collaborative monitoring over broad scales and levels of ecological organization can inform conservation efforts necessary to address the contemporary biodiversity crisis. An important challenge to collaborative monitoring is motivating local engagement with enough buy-in from stakeholders while providing adequate top-down direction for scientific rigor, quality control, and coordination. Collaborative monitoring must reconcile this inherent tension between top-down control and bottom-up engagement. Highly mobile and cryptic taxa, such as bats, present a particularly acute challenge. Given their scale of movement, complex life histories, and rapidly expanding threats, understanding population trends of bats requires coordinated broad-scale collaborative monitoring. The North American Bat Monitoring Program (NABat) reconciles top-down, bottom-up tension with a hierarchical master sample survey design, integrated data analysis, dynamic data curation, regional monitoring hubs, and knowledge delivery through web-based infrastructure. NABat supports collaborative monitoring across spatial and organizational scales and the full annual lifecycle of bats.

## Introduction

**Fig. 1:**
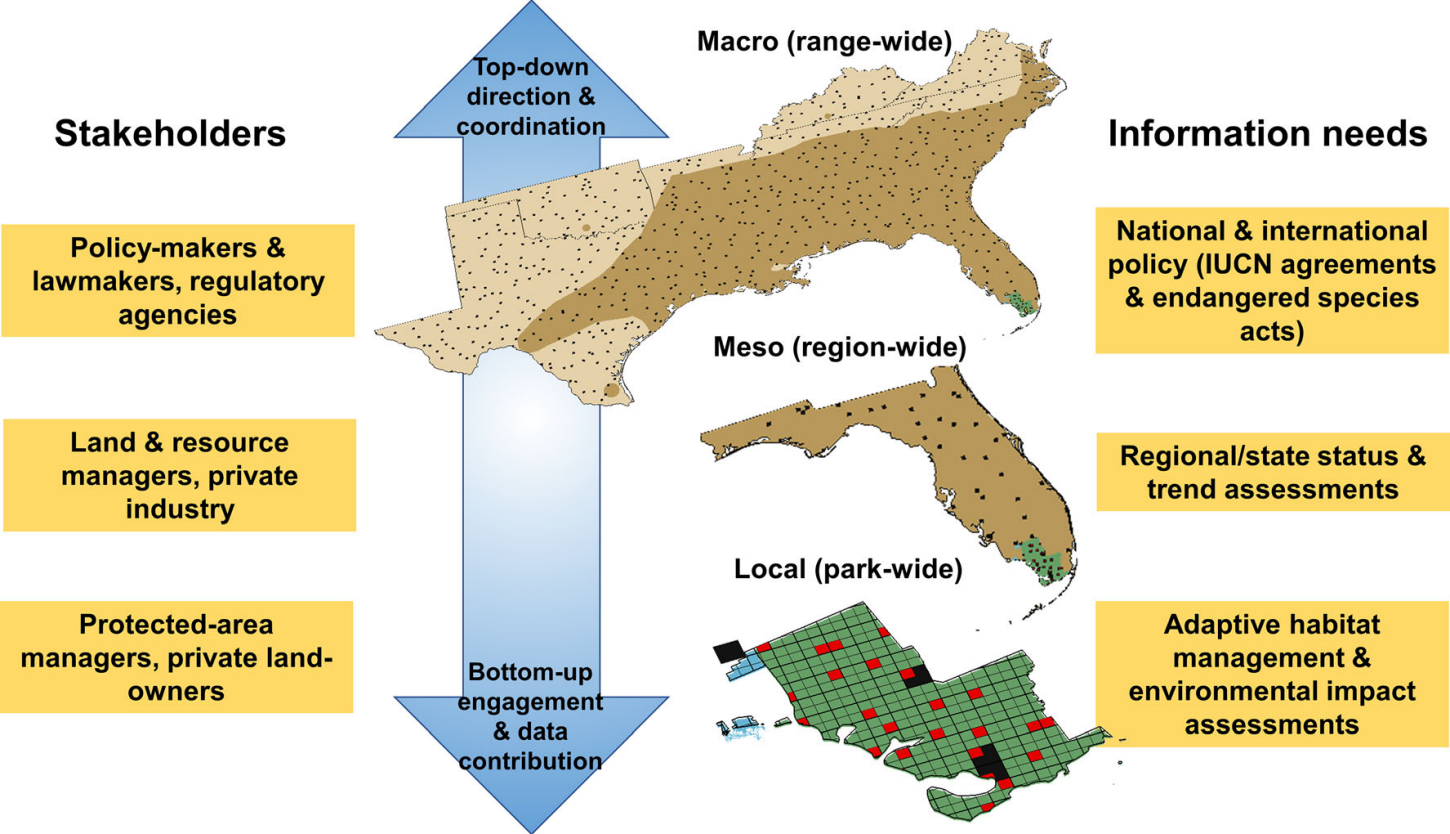
The range of the Seminole bat (*Lasiurus seminolus*) across southeastern U.S. with 3% sampling intensity of the master sample (black grid cells) (macro-scale), the grid overlay for the state of Florida (meso-level), and intensified sample across Everglades and Big Cypress National Parks and Florida Panther and Ten Thousand Islands National Wildlife Refuges protected-area jurisdictions (local-scale). Within the protected areas, four NABat grid cells (black fill) from the 3% range-wide target sample are combined with a randomized subsample of 5-km × 5-km grid cell subunits (red fill). Data improve inferences across the protected areas and contribute to regional and range-wide analyses

Preserving the natural heritage of Earth requires informed conservation action operating over multiple, often hierarchical, jurisdictions among a wide array of constituencies (Wyborn and Bixler 2013). The information needed for appropriate and efficient allocation of resources for management and conservation can be obtained through monitoring (Nichols and Williams 2006). Given the extent and magnitude of the modern biodiversity crisis and limited resources available to address it, acquiring information through monitoring is often best attained through a collaborative framework. Collaborative monitoring distributes the logistical and financial burden of data collection and helps engage the community of conservation actors (Danielson et al. 2017; Steenweg et al. 2017). A pressing challenge is that the conservation community is often interested in information at different (often nested) spatial extents (Fig. 1) and at different temporal life stages (Hostetler et al. 2015). Multinational organizations (e.g., International Union for the Conservation of Nature (IUCN) and European Environmental Agency) and federal and state/provincial governments require information on the status and trends of species at range-wide or jurisdictional scales and across their full annual lifecycle to inform regulatory decisions. State/provincial governments, the private sector, joint ventures (e.g., North American Waterfowl Management Plan, (Patterson 1995)), and other regional conservation partnerships (e.g., Midwest Landscape Initiative^1^) use best available science at meso (regional) scales to assess consequences of anthropogenic stressors and inform business or management strategies. At local scales, municipalities, parks, and protected areas require information on habitat use and trends to determine the potential impact of local actions. Collating the requisite data across a collaborative monitoring network at each of these scales of organization and disseminating information back to the wide array of interested parties is a profound challenge in monitoring.

There are at least two ways to organize monitoring (Bennun et al. 2005). Monitoring programs in which actions and policies are initiated at the highest level and provided to those charged with data collection are often described as systems of top-down control. These prescriptive systems are characterized by rigorous application of protocols, sample designs, and data curation to ensure standardized data acquisition and management (e.g., USDA Forest Service, Forest Inventory and Analysis Program; Bechtold and Patterson 2005). Conversely, bottom-up organization often arises in response to the need for local information. For example, the National Park Service’s Inventory and Monitoring Program (Fancy et al. 2009) permitted distributed networks of parks to identify park-relevant “vital signs.”

Both approaches have their limitations. Top-down monitoring efforts may suffer from too little flexibility to meet the needs of the constituents gathering the data. For example, information obtained from broad-scale monitoring efforts can lack relevancy at local scales, resulting in declines in participant retention and lack of motivation for data submission (Pocock et al. 2015). Conversely, information obtained from bottom-up monitoring efforts to inform range-wide species’ status and trend assessments can be problematic due to a lack of common objectives, adherence to a unifying sample design, and mismatches in the form of data and manner in which they were collected (Field et al. 2007; Conrad and Hilchey 2011). As a result, bottom-up initiatives can lead to a dataset of varying quality with data collected following a variety of protocols with uncertain utility for rigorous assessment at larger scales (Matsuoka et al. 2014).

Successful multi-scale collaborative monitoring programs should adequately address three important questions: (1) How does the monitoring program remain relevant for satisfying information needs at a variety of spatial scales? (2) How can participation and collaboration be motivated and sustained over the spatial extent of the domain of interest? (3) How is scientific rigor assured without the rigor mortis imposed by overly prescriptive control? Here, we present the collaborative monitoring framework of the North American Bat Monitoring Program (NABat) as a model approach for addressing these challenges.

## NABat: North American Bat Monitoring Program

Bats play important roles in maintaining healthy ecosystems (Kunz et al. 2011) and have significant economic benefits to agriculture (Boyles et al. 2011; Taylor et al. 2018) and tourism (Bagstad and Widerholt 2013). In North America, bat populations face multiple continuing and emerging threats (O'Shea et al. 2003). Initiated in 2015, NABat is a collaborative, long-term monitoring program designed to assess the status and trends of North American bats at local, regional, and range-wide scales (Loeb et al. 2015). The overarching programmatic goal of NABat is to provide regular assessments on the status and trends in abundance (e.g., Thogmartin et al. 2012) and distribution (e.g., Rodhouse et al. 2019) of North American bat species, while also meeting local and regional stakeholder information needs (e.g., informing forest management practices) (Loeb et al. 2015). Analytical products on long-term viability of bat populations across North America delivered by NABat can inform effective conservation decision-making by tailoring information to meet the needs of different stakeholders (Fig. 1).

**Fig. 2:**
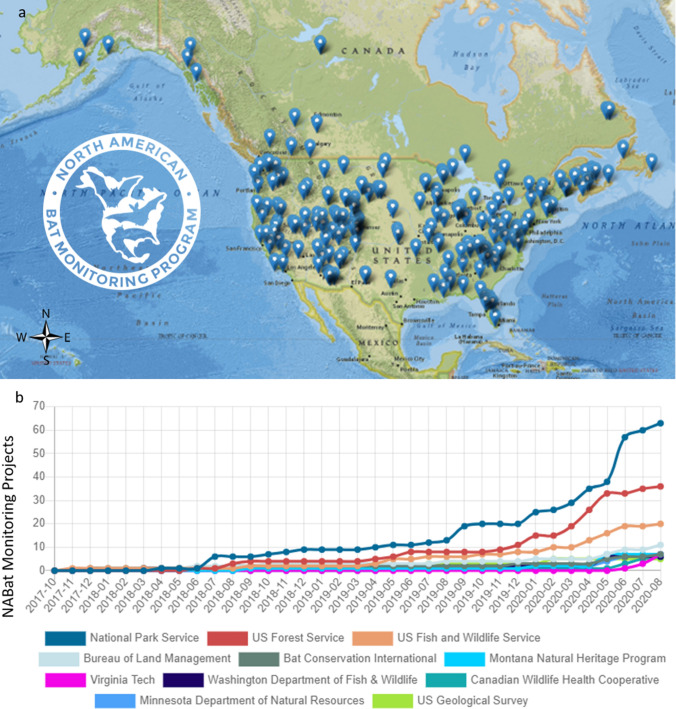
a. Map of the NABat monitoring network (available at https://sciencebase.usgs.gov/nabat/#/home/explore-nabat-data, accessed on 8/18/2020). Blue points represent locations of NABat monitoring projects as documented by users of the NABat website (www.nabatmonitoring.org). b. Number of registered users over time for the 11 organizations with the most registered users (https://sciencebase.usgs.gov/nabat/#/home, accessed on 8/20/2020)

NABat has worked to bring together a growing network of stakeholders concerned with the conservation of North American bats in the United States and Canada with aims to eventually include the mega-diverse country of Mexico (Figs. 1 and 2). An implementation strategy for NABat was initially outlined in “A Plan for the North American Bat Monitoring Program (NABat)” including ideas about how the program might be structured into the future (Chapter 10) (Loeb et al. 2015). Since then, the multi-organizational NABat Planning Core Team co-led by representatives of the U. S. Geological Survey (USGS), U. S. Fish and Wildlife Service, National Park Service, USDA Forest Service, Bureau of Land Management, Canadian Wildlife Service, United States Department of Defense, Bat Conservation International, Wildlife Conservation Society Canada, and Canadian Wildlife Health Cooperative have provided national and international programmatic support as well as scalable solutions that improved efficiencies in monitoring activities across multi-jurisdictional boundaries. With input from the NABat Core Planning Team, state/provincial and regional monitoring coordinators and working groups, USGS has provided overall program coordination, training and monitoring tools, data management, IT infrastructure, research and development of statistical methods, and analytical support for species status and trends assessments^2^.

At the regional level (one or more state or province), monitoring efforts have been coordinated and/or conducted by state, provincial, federal, and tribal land resource management and conservation agencies, universities, and non-governmental conservation organizations through a growing number of regional monitoring hubs (see *The Master Sample in Action: Regional Monitoring Hubs*). Other important contributors include environmental consultants, local naturalist groups, private industry (e.g., Duke Energy), and trained citizen scientists. These partners have utilized the NABat’s probabilistic sample design, monitoring guidance, and data management tools to contribute standardized bat population data (Fig. 1). They have also provided critical feedback through regional and technical working groups that helped to refine monitoring methods, analytical approaches, data submission pathways, and reporting mechanisms.

NABat blends top-down direction with bottom-up flexibility for addressing common and diverse information needs across multiple organizational scales. Top-down direction includes working with regional and local NABat partners to establish and maintain data collection efforts following the program’s sampling framework and data standards^3^. When monitoring efforts of local partners are spatial and/or temporal subsets of larger assessments, collaborative monitoring based on the NABat sample design can reduce the need for intensive local survey efforts. The need for bottom-up flexibility can arise when additional information is required to address local management questions. In this case, local-scale studies can be nested within the NABat grid-based master sample. Importantly, NABat has also developed data management workflows (Banner et al. 2018) and statistical procedures to integrate complimentary survey data collected outside the program’s sampling framework (Irvine et al. 2018).

NABat strives to improve the state of conservation science for North American bats, which are currently experiencing a variety of threats including disease, expansion of wind energy, and rapid habitat loss and fragmentation (O’Shea et al. 2016). The most severe threat is currently the white-nose syndrome (WNS), a disease caused by an invasive fungal pathogen (*Pseudogymnoascus destructans*) (Lorch et al. 2011; Warnecke et al. 2012). WNS has caused rapid and severe declines of several species of hibernating bat over the past decade since it was first discovered (Frick et al. 2010; Langwig et al. 2012; Thogmartin et al. 2012; Frick et al. 2015). It is now confirmed in at least 11 species of bats across 35 states and seven Canadian provinces^4^. In addition to the threat of WNS, fatalities of bats at wind energy facilities could also lead to severe and rapid population declines for migratory bats, as has been hypothesized for hoary bats (*Lasiurus cinereus*) (Frick et al. 2017). Impacts from WNS and wind energy development may be compounded by other long-term and chronic stressors (e.g., direct human disturbance and pollution) (O’Shea et al. 2016). Interestingly, some species appear to be shifting and possibly expanding their ranges, including the Brazilian free-tailed bat (*Tadarida brasiliensis*) (McCracken et al. 2018; Irvine et al. 2020). Broad-scale long-term monitoring to assess changes in species relative abundance and distributions in response to environmental drivers is key to developing and evaluating effective conservation and management strategies for these highly dynamic bat communities (Loeb et al. 2015; Frick et al. 2019).

## The master sample concept: A catalyst for collaborative monitoring

The master sample concept describes the process of enumerating, in random order, all sample units within a finite sampling frame, e.g., grid, to support flexible, probabilistic environmental surveys across broad geographic extents (Box 1; Larsen et al. 2008). The concept was first proposed in the mid-twentieth century for agricultural purposes (King 1945) and has re-emerged with the development of spatially balanced randomization algorithms (Larsen et al. 2008). The master sample concept provides a framework for facilitating collaborative monitoring among multiple partners (Larsen et al. 2008). A fundamental property of the spatially balanced master sample is the exhaustive random ordering of all sample units covering the entire geographic area based on the generalized random-tessellation stratified (GRTS) design. Following the GRTS sampling order ensures that any ordered spatial subset of units (e.g., all units with greater than 0% land owned by the U.S. Forest Service in the state of Colorado) remains spatially balanced with known inclusion probabilities (Stevens and Olsen 2004). By selecting the highest priority grid cells within their jurisdictional boundary, partners can scale their level of effort (i.e., subsample size) commensurate to available resources, yet still contribute to a larger statistically valid probability-based sample for regional or range-wide analyses and reporting (see Figs. 1 and Box 1).

**Box 1:** The master sample approach of the North American Bat Monitoring Program (NABat). GRTS = generalized random-tesselation stratified (GRTS)

North American Bat Monitoring Program (NABat) utilizes a grid-based sampling frame for the continental US, Canada, and Mexico. The NABat grid is composed of 10-km×10-km grid cells as sample units (denoted as Unit ID, Panel 1a). The GRTS algorithm was applied to the entire NABat grid which results in an ordered list of sample units (denoted as GRTS Order, Panel 1a) that are attributed by jurisdiction (for example, USFS for U.S. Forest Service land, and State to denote state managed lands,). As a simple example of how to use the GRTS ordered master sample list, within a common spatial domain (e.g., the state of Colorado; Panel 1b), a state wildlife agency could survey unit 12, 26, 103, 87, and 786 and the USFS could survey 111, 289, 115, and 17. A greater sample size is acheived by coordinating among partners to identify those grid cells that can be shared but only need to be surveyed by one group. The sample units with the lowest GRTS order within a given spatial domain are considered the “highest priority” to survey. The number of grid cells to survey should be informed by power analyses (e.g., Banner et al. 2019).	
	*Example. Increasing statistical power by coordinated sharing* Since 2015, six federal, state, and local partnering organizations have monitored 110 grid cells across Colorado, USA. Colorado Parks and Wildlife (CPW) and Colorado Natural Heritage Program (CNHP) have led the collaborative effort by surveying up to 51 grid cells per year following NABat stationary acoustic monitoring data collection protocols. CPW and CNHP selected grid cells by first sub-setting the NABat master sample to all grid cells that overlapped Colorado (n = 2811). Based upon power analyses of NABat survey data (Banner et al. 2019), 3% of the grid cells were designated as high priority (84 of 2811). Of the top 84 priority grid cells, 55 were selected for survey (52 by CNHP, 2 by neighboring state partners, 1 by US Geological Survey), while 32 were high priority but not selected because of a lack of access or resources. The USDA Forest Service (USFS) manages more land than any other entity in Colorado. Of the 55 high priority grid cells surveyed, 17 grid cells overlapped USFS lands; and of the 29 priority grid cells not surveyed, 8 intersect USFS lands. If USFS joins partners in implementing NABat in Colorado, their minimum survey effort requirement is only the 8 unsampled grid cells intersecting USFS lands (blue cells, Panel 1b), not the full 25 grid cells. Once other partners ‘adopt’ the remaining 21 priority grid cells, state-wide information on the probability of species-specific occurrence will be greatly improved. The master sample design established by NABat allows for data collected by multiple agencies to be used to make inference to every grid cell within Colorado using spatial predictions, regardless of ownership and survey status. The collaborative framework of NABat leverages resources across partnering organizations, developing a network for shared stewardship of our natural resources. As such, individual partners, such as the USFS in Colorado, benefit form minimal investment.

An important feature emerging from master sample implementation is the identification of shared sample units among jurisdictions. Building and maintaining IT infrastructure for tracking and communicating the status of shared sample units reduces redundancy and facilitates collaboration and economy of scale (Box 1). The flexibility of the master sample applied to a continental finite sampling frame (e.g., a grid) supports capability to scale up data contributions from surveys done across many jurisdictional subsets of the master sample. The approach also allows for contributions from compatible data collected outside of the formal master sample, including legacy monitoring programs (Irvine et al. 2018). An opportunity not yet fully realized is to use the NABat master sample for recasting the count-based surveys into a common probabilistic framework with summertime bat acoustic surveys conducted for tracking species distribution, range expansion, and new species occurrence. Efficient data management practices that enable tracking and retrieving contributor information on their master sample implementation are crucial for enabling data synthesis and integration required to achieve range-wide inferences.

## Modern tools support top-down direction while motivating participation

NABat has developed a web-based application (‘NABat Partner Portal,’ 5) and centralized, online database to help direct monitoring efforts while generating local participation in its collaborative monitoring framework^6^. The application provides access to the NABat Master Sample (Talbert and Reichert 2018) (Box 1) via the ‘Grid Cell Selection Tool.’ Through this mapping tool, users filter the NABat Master Sample to their jurisdiction (state, province, region, or by landownership). Within their jurisdictional subset of the NABat Master Sample, users follow the predetermined GRTS sampling order to ‘select’ grid cells (sample units) to be surveyed. If a user determines a grid cell is not accessible to be surveyed, the user documents this evaluation in the tool and selects the next available grid cell following the predetermined GRTS sampling order. Once a cell has been selected, the user specifies at least one type of monitoring method (*see* “Multiple lines of evidence for full annual life cycle understanding”). NABat partners are expected to survey the same grid cells annually following national guidance (Loeb et al. 2015) and regionally specific standard operating procedures (e.g., Rodriguez et al. 2019). Power analyses tailored to regional specifications guide the required survey effort needed to achieve measurable objectives for estimating status and trends (Banner et al. 2019). If partners are no longer able to survey a cell, (lack of resources, retirement, etc.) they use the tool to un-select the grid cell, which makes it available for others to select and survey that year. The application also allows users to share contact information among potential collaborators. This depth of information helps minimize duplicative efforts and identify synergistic research opportunities. Steering partners within a region to survey the highest priority (ranked) sample units following a unified probabilistic sample design (the NABat Master Sample) permits straightforward pooling of data. NABat intends to use pooled data for rigorous statistical analyses to address information needs at the local (jurisdictional), regional, and range-wide scales (e.g., Rodhouse et al. 2019). Integrating partner contributed datasets reduces the level of effort and associated cost for an individual partner while providing the collective with improved model estimates of species’ status and trend. Cost-effective solutions help motivate participation in collaborative monitoring efforts.

## The master sample in action: Regional monitoring hubs

**Fig. 3:**
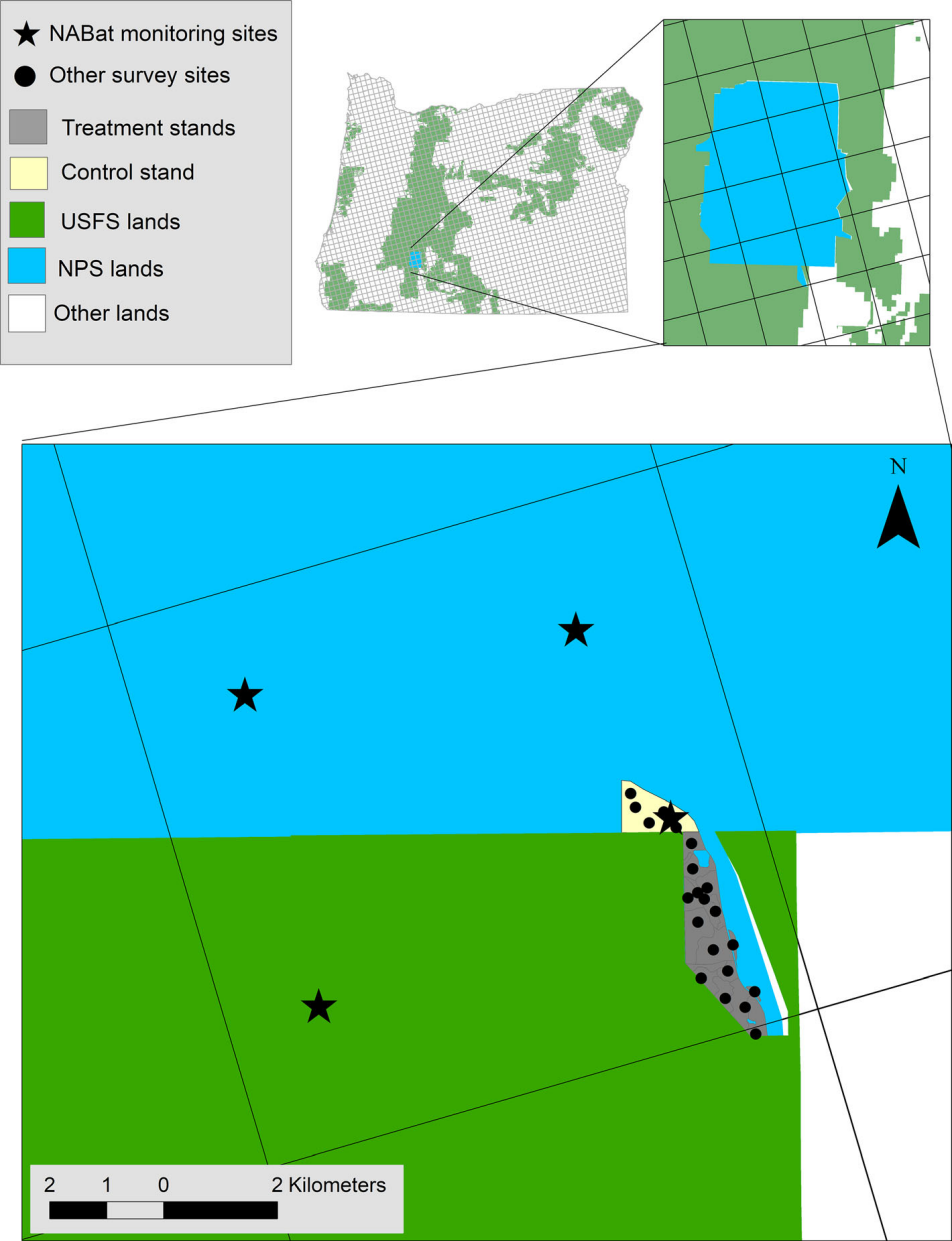
In Crater Lake National Park, Oregon, USA, a local-scale study of the effects of forest thinning for fuels reduction was nested within the NABat grid-based master sample. Four replicate survey locations for NABat summertime acoustic surveys (stars) include one within the study area. Additional replicate survey locations within the study area (black circles) were placed within forest treatment and control stands. This example highlights the scalability of the NABat program in a way that helps bridge the top-down and bottom-up relevancy gap

NABat is delivered through an increasing number of monitoring hubs—networks of local partners across one or more states/provinces. These hubs help to maximize efficiencies in resource allocation, standardize data collection protocols, provide training, and contextualize monitoring data to address needs of local partners. NABat monitoring hubs are organized through an entity (e.g., university, state agency, or non-governmental organization). This entity coordinates members of the hub to ensure that data collection efforts throughout the region follow the predetermined GRTS sampling order of the NABat Master Sample. Coordinated efforts through the hub help prevent redundancies, facilitate equipment sharing, provide training, and streamline data submission and analysis (e.g., the Northwestern Bat Hub at Oregon State University-Cascades, (Rodriguez et al. 2019)). When partners have monitoring objectives that require additional localized data collection, such as those required for regulatory purposes, these efforts supplement the broader regional synthesis (Fig. 3). Regional hubs strengthen local human networks and increase bottom-up engagement.

## Multiple lines of evidence for full annual lifecycle understanding

**Fig. 4:**
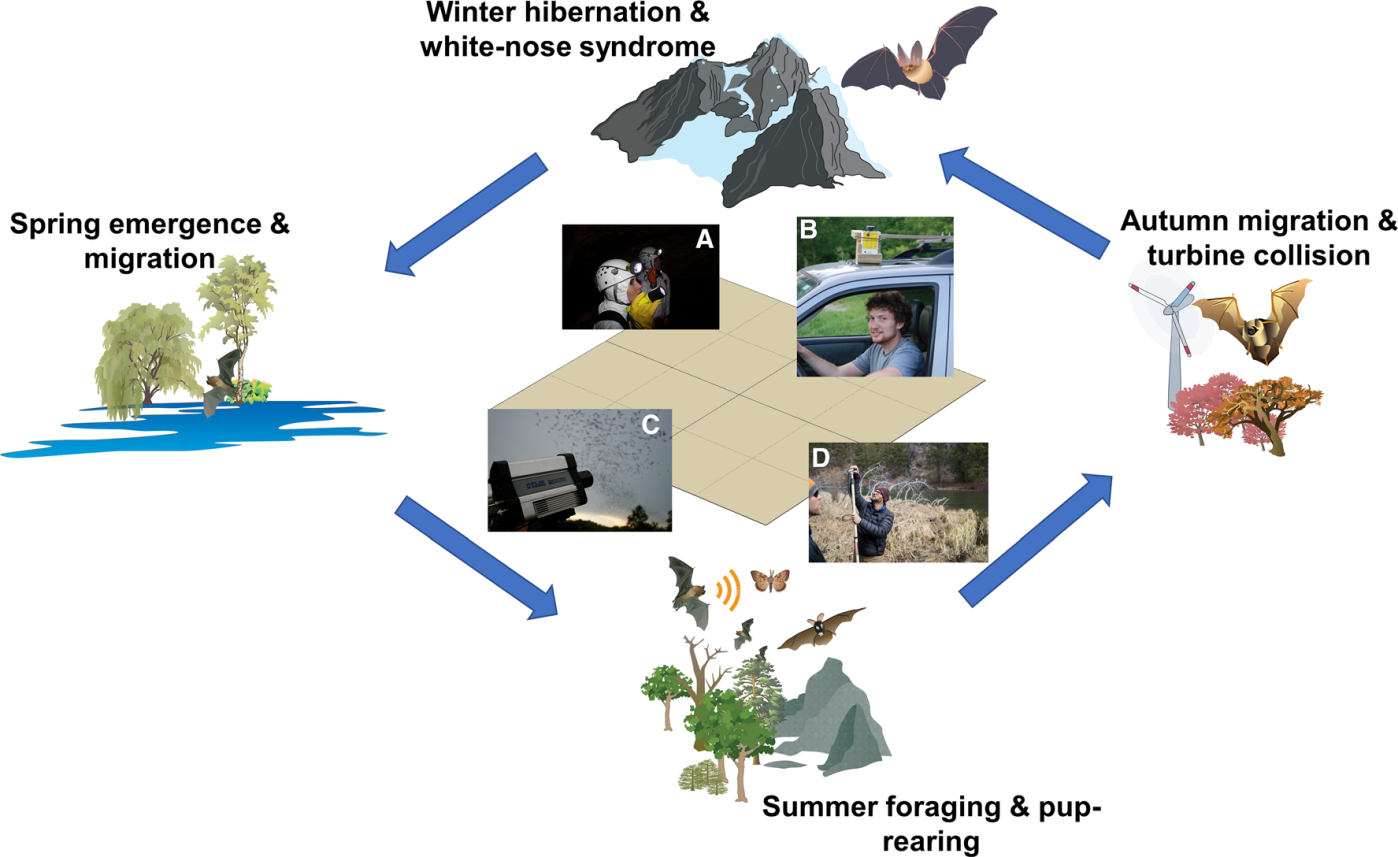
The North American Bat Monitoring Program uses multiple methods to survey for multiple bat species at multiple points throughout the annual lifecycle of a bat. The hierarchical grid-based master sample (represented at center) provides the common architecture to facilitate this. A. Observers count bats and conduct surveillance for white-nose syndrome at a winter hibernaculum. Image courtesy of Gary Peeples, used with permission. B. Acoustic recording equipment attached to a vehicle for a mobile acoustic transect survey for bats. Image courtesy of Michael Whitby, used with permission. C. Camera-based count survey of brazilian free-tailed bats (*Tadarida brasiliensis*) emerging from a roost. Image courtesy of Ann Froschauer. D. Setting up a stationary acoustic detector to monitor bats. Image courtesy of Oregon State University. Artwork courtesy of the Integration and Application Network, University of Maryland Center for Environmental Science (ian.umces.edu/symbols/)

Bats are highly cryptic and exhibit a variety of life histories and survival behaviors that make them difficult to observe (Barlow et al. 2015). As such, no single data collection method is adequate for monitoring all 47 species of bats found in the United States and Canada. NABat employs five survey methods (summer stationary acoustic monitoring, summer mobile acoustic monitoring, internal summer maternity colony counts, external counts of bats emerging from summer roosts, and winter colony counts) (Fig. 4). These survey methods provide multiple lines of evidence that help to refine understanding of the status and trend of individual species.

For example, understanding the status of northern long-eared bats (*Myotis septentrionalis*) is enhanced by sampling in both the summer and winter seasons. Rapid declines in numbers of observed hibernating northern long-eared bats at winter roosting sites after the emergence of WNS (Langwig et al. 2012) led to the species being listed as Threatened under the Endangered Species Act in 2015. Northern long-eared bats disappeared entirely within 4 years at 69% of known hibernacula in the eastern United States (Frick et al. 2015). However, summer surveys (including acoustic sampling) suggested remnant populations of northern long-eared bats persist in these regions (G. Turner, PA Game Commission, C. Herzog, NY Department of Conservation, pers. comm. 1/16/2020). Some proportion of the population likely uses unknown sites during hibernation and detections of northern long-eared bats during summer surveys may help identify remnant populations. A full appreciation of the true population-level impacts of WNS on northern long-eared bats therefore benefits from both summer acoustic surveys and winter colony counts.

NABat uses data from both count-based surveys and acoustic recordings of echolocating bats to assess population status and trends (Fig. 4, Loeb et al. 2015). Counts of bats at winter roost sites represent the best opportunity for estimating abundance for some species but are inadequate for depicting the range of these hibernating species because winter roost sites are a spatially restricted subset of the occupied landscape. Further, counts of hibernating bats are an important source of data for determining population trends, but are only useful for the subset of species aggregating in medium–high densities during winter (Weller et al. 2009). For most of the bat species that NABat intends to track, annual counts at hibernacula are not available, requiring alternative means of status determination. Acoustic recordings of bat echolocations from summer stationary acoustic monitoring can be classified to species and used in an occupancy modeling framework (accounting for imperfect detection of each species) to describe the area over which a species occurs and for trends in occupancy over time (MacKenzie et al. 2002; Royle and Kéry 2007). However, acoustic data collected from stationary acoustic monitoring (e.g., multiple detectors deployed over multiple nights) can only provide an oblique understanding of population size (e.g., Rodhouse et al. 2019; Wright et al. 2020). In comparison, acoustic data collected from summer mobile acoustic monitoring along road transects can be used to estimate population trends via assumed individual bat calls (Roche et al. 2011; Whitby et al. 2014; Barlow et al. 2015). Yet, species detection rates from mobile acoustic surveys can be markedly lower compared to stationary acoustic monitoring (Whitby et al. 2014) such that data may be insufficient for monitoring some species. Given limitations in monitoring methods and complexities of bat life history, NABat has been working to integrate multiple lines of evidence from both summer and winter seasons, which should improve accuracy in describing species status. Analytical methods that integrate data across the annual lifecycle are increasingly available (Hostetler et al. 2015).

## Tailored designs meet partner-specific information needs

A benefit of the master sample is that partners can construct a tailored design for their specific spatial domain and information needs. For example, in North Carolina, USA, acoustic monitoring of bats following NABat guidance for mobile transect surveys (Loeb et al. 2015) were conducted annually from 2015 through 2019. Each year, 45 transects were surveyed by at least 20 different partnering organizations (Li et al. 2019). Each partner was only responsible for surveying transects within the highest priority grid cells within their respective jurisdiction or area of interest. Then, data were sent to a regional hub where processing of acoustic recordings was standardized and cost-effective. Importantly, timely reports and state-wide assessments were provided, and partners had access to each other’s data based on agreed upon data-sharing policies. This information-sharing incentivized continued participation by partners. NABat facilitates controlled access to monitoring data through a data request submission processes^7^.

All participants in NABat monitoring in North Carolina were interested in contributing local monitoring data to range-wide species assessments, but partners also had a variety of additional information needs. Government agencies required inventory information of local species for conservation decision-making and industry participants needed similar information to fulfill legal compliance requirements. In addition, non-profit organizations and trained citizen scientists adopted transects for public education purposes. Research scientists from academic institutions used resulting data to investigate questions related to environmental stressors, ecological scaling, and niche partitioning (Schimpp et al. 2018; Li et al. 2019). Thus, NABat offered a collaborative sampling framework for gathering reliable information to meet each partner’s needs.

## Addressing information needs at multiple scales

Local information needs are not always met through top-down, standardized monitoring, sometimes leading to the demise of such efforts (Fancy et al. 2009). When funding to support efforts is reduced, data collection efforts that appear uninformative for addressing the needs of local resource managers can be temporarily or permanently eliminated (Fancy et al. 2009). Initial development of NABat was designed to accommodate scalable effort and allows for combining compatible data for broader geographic syntheses (Loeb et al. 2015; Irvine et al. 2018).

For example, in Crater Lake National Park, Oregon, local engagement with NABat was motivated through integration of a study of bat community response to forest wildfire fuels reduction (thinning; Fig. 3). This local-scale study was nested within the grid-based sampling frame such that a portion of the acoustic survey data could also contribute to regional and range-wide NABat trend assessments. Challenges included enforcing compatible methodology during sighting and deployment of recording devices and in data management. The study area was predetermined by existing land management priorities and boundaries and, therefore, did not occur within a high priority (i.e., by following GRTS order) NABat grid cell. Despite monitoring not occurring within state-wide priority cells, the resulting data were useful when pooled with the regional data for estimating population change (Rodhouse et al. 2019). Results suggested that hoary bat populations may have declined throughout the northwestern United States (Rodhouse et al. 2019). The project also proved an effective way to motivate local-scale (park) engagement with NABat.

## Early successes and ongoing challenges

In less than 5 years, NABat has grown to more than 500 online registered users, with data collected across 49 U.S. states and 6 Canadian provinces. In 2020 alone, the NABat database increased from 29 742 database records to more than 44 000 000 records. NABat now facilitates controlled access to these monitoring data through an online data request process^8^. In these initial years, NABat has used monitoring data to advance methods, such as develop extensions of occupancy models to account for false-positive species detections from acoustic recordings (Banner et al. 2018; Wright et al. 2020) and inform approaches using stationary monitoring (Wright et al. 2016; Banner et al. 2019) that are useful for the core purpose of NABat but contribute broadly to statistical approaches and methodologies using acoustic detections. NABat data have also been used to assess summer and winter distributions and establish baseline species status and trends in several regions (e.g., Neece et al. 2018; Meierhofer et al. 2019; Rodhouse et al. 2019; Irvine et al. 2020),

NABat provides a model for efficient monitoring of wildlife informing shared stewardship of our natural resources that may be useful for developing similar efforts for monitoring bats (and other taxa) in other parts of the world. National monitoring programs for bats are not common outside of Europe, yet share some similarities to NABat in that they often incorporate and use different data types, such as acoustic detections during summer and winter colony counts, and have some elements of a multi-scale approach by adhering to general guidance provided by the Eurobats Secretariat (Battersby 2010). Many of these national-scale programs (e.g., British Bat Monitoring Programme) evolved from bottom-up approaches and rely on volunteers and citizen-science (Battersby 2010; Barlow et al. 2015). As such, they are often based on convenience sampling where citizens collect monitoring data from acoustic or emergence count surveys without using a probabilistic sampling design (Battersby 2010). Compared to NABat, the national monitoring programs in European countries operate over much smaller spatial extents and often leverage long-standing public interest in bats and citizen engagement in data collection and conservation more broadly (Battersby 2010; Barlow et al. 2015).

In contrast, NABat currently relies almost entirely on trained wildlife professionals for data collection efforts who often have greater access to restricted locations (e.g., private property, caves, and mines). One hurdle to building broader engagement of community scientists in the NABat effort is in providing timely results to potential community participants, which is currently limited by the cost and time-delay required for processing acoustic data. Recording and classifying the echolocation calls to a species requires specialized and often expensive equipment and software (Reichert et al. 2018). Following the NABat Master Sample to select monitoring locations may require partners to travel to inconvenient or remote locations rather than deploy detectors nearby their homes and offices. Also, the reality of a probabilistic sampling design is that some prioritized locations may not be highly suitable habitats for bats, but these data are still useful. In fact, the data about conditions in which bats are not observed or detected (non-detections) are as valuable as documenting where they do occur (detections) in space and time. These constraints require that participants are motivated, open to learning new techniques, and financially capable of carrying out NABat monitoring activities.

In Mexico, the government supports an ambitious program to monitor biodiversity. The National Biodiversity Monitoring System (SNMB^9^) collaborates across several of Mexico’s federal agencies responsible for natural resource management (Garcia-Alaniz et al. 2017). The SNMB project includes biodiversity monitoring for many taxa, including acoustic monitoring for bats. Mexico is a mega-diverse country with high species richness of bats and until recently there were not extensive acoustic libraries of echolocation calls of bats (Zamora-Gutierrez et al. 2016, 2020). One challenge for NABat will be building a collaborative integration with these existing monitoring efforts in Mexico, especially for wide-ranging species whose distributions overlap geo-political boundaries or migrate trans-nationally.

## Conclusions

NABat provides an extensible framework appealing to coarse, intermediate, and fine scales of organizational hierarchy, spatial extent of operation (jurisdictional mandates), and information needs. Collaborating partners can apportion the NABat master sample across their land management jurisdiction to meet local needs while displaying “selected cells” to inform regional partners of extant survey effort. Gaps in regional-scale survey effort can then be targeted to increase coverage of high-priority units and increase statistical power. A probabilistic master sample design with an accompanying centralized database for both the sampling design and resulting data ensures defensible data integration to inform range-wide species assessments.

As technological tools for monitoring bats become more widely available and easily deployable across all seasons, the increasing data volumes and acquisition rates will require new collection, integration, data transfer and storage, and synthesis strategies. Scaling and integration of multiple data types that are seasonally misaligned will require new statistical approaches. The NABat program foresees these challenges and is building the infrastructure to meet them.

Given limited resources, aligning local conservation and resource management objectives with important continental-scale priorities is essential for a successful long-term conservation program. NABat provides an important and efficient bridge between local, regional, and continental scales. When objectives differ across conservation and management scales, NABat’s design can satisfy local information needs while providing data to species status and trend assessments at regional and continental scales, thus achieving an economy of scale for conservation monitoring.

## Footnotes

1 http://www.mafwa.org/?page_id=3391.

2 www.nabatmonitoring.org.

3 https://www.nabatmonitoring.org/resources.

4 www.whitenosesyndrome.org.

5 https://sciencebase.usgs.gov/nabat/#/home.

6 Available at https://www.nabatmonitoring.org/.

7 https://sciencebase.usgs.gov/nabat/#/home/explore-nabat-data.

8 https://sciencebase.usgs.gov/nabat/#/home/explore-nabat-data.

9 https://www.biodiversidad.gob.mx/monitoreo/monitoreo-biodiversidad/.
